# Prudence, Emotional State, Personality, and Cognitive Ability

**DOI:** 10.3389/fpsyg.2016.01688

**Published:** 2016-10-28

**Authors:** Adriana Breaban, Gijs van de Kuilen, Charles N. Noussair

**Affiliations:** ^1^Department of Economics, Tilburg UniversityTilburg, Netherlands; ^2^Department of Economics, University of ArizonaTucson, AZ, USA

**Keywords:** emotions, prudence, personality, cognitive ability

## Abstract

We report an experiment to consider the emotional correlates of prudent decision making. In the experiment, we present subjects with lotteries and measure their emotional response with facial recognition software. They then make binary choices between risky lotteries that distinguish prudent from imprudent individuals. They also perform tasks to measure their cognitive ability and a number of personality characteristics. We find that a more negative emotional state correlates with greater prudence. Higher cognitive ability and less conscientiousness is also associated with greater prudence.

## Introduction

The study of the role of risk preferences in decision making has primarily focused on the implications of risk aversion, i.e., the preference for a certain payment to a lottery with the same expected value. If one assumes that individuals maximize expected utility (e.g., for prescriptive applications), risk aversion implies that the utility function for money is concave (i.e., that u”(x) < 0). However, empirical work has shown that the degree of risk aversion is often affected by psychological factors not captured by the expected utility model, such as the perceived likelihood of events and the perceived domain of the outcomes (e.g., Tversky and Kahneman, 1992). Moreover, theoretical work has shown that risk aversion is not the only facet of preference governing economic decision making: it is becoming increasingly recognized that the higher order risk attitudes of *prudence* and *temperance* complement the role of risk aversion in economic decision making in important ways. For example, in the realm of saving behavior, while risk aversion drives the preference to smooth consumption over time (*consumption smoothing*; Friedman, 1957), prudence determines how saving behavior changes as future income becomes riskier (*precautionary saving*; Kimball, 1990). Other examples of areas of economics, in which higher order risk preferences have been found to play an important role in influencing behavior, include bidding in auctions (Esö and White, 2004), bargaining (White, 2008), tax compliance (Alm, 1988), and rent seeking (Treich, 2010).

Within the expected utility framework, prudence is typically defined as the convexity of marginal utility (u”'(x) > 0), while temperance is equivalent to a negative fourth derivative of the utility function (u””(x) < 0). However, Eeckhoudt and Schlesinger (2006) have introduced behavioral definitions, based on observable revealed preferences, of prudence and temperance that are model-free in the sense that they retain validity if expected utility fails to accurately describe choice behavior (e.g., see Starmer, 2000). The definitions of Eeckhoudt and Schlesinger (2006) are based on risk apportionment. In particular, a decision maker (DM) is prudent if she prefers to apportion an unavoidable zero-mean risk to a relatively high rather than to a low wealth state, while a temperate DM prefers to apportion two independent zero-mean risks across different states of nature.

Several recent papers have used the behavioral definitions of Eeckhoudt and Schlesinger (2006) to quantify higher-order risk preferences empirically. The results from these studies show that the degree of prudence varies considerably among individuals within the population (Deck and Schlesinger, 2010, 2014; Ebert and Wiesen, 2011, 2014; Noussair et al., 2014), though all of these studies agree that a majority of individuals are prudent. Furthermore, Noussair et al. (2014), who study a large sample of demographically representative individuals, find that those who exhibit more prudent decision making also have greater savings, lower debt, more wealth and higher educational attainment. The results for the prevalence of temperance within the population are more mixed (e.g., Deck and Schlesinger, 2010, 2014; Noussair et al., 2014).

It is also widely recognized in behavioral economics, psychology, and management, that there is an important connection between emotional state and risk preferences. However, research in this area has focused exclusively on the link between emotional state and risk aversion. This research can be classified based on whether it considers the relationship between risk taking and overall valence (positivity or negativity of emotional state), or to specific emotions such as fear, anger, and happiness, as correlates of decision making. Johnson and Tversky (1983) propose that a positively-valenced emotional state increases risk taking, because it makes beliefs about outcomes more optimistic. This relationship is termed the *Affective Generalization Hypothesis*. On the other hand, Isen et al. (1988) have argued that a positive mood leads to less risk taking because individuals wish to preserve the positive emotional state and insulate themselves from negative outcomes. This is referred to as the *Mood Maintenance Hypothesis*.

In addition to overall valence, specific emotions have been associated with risk taking. The Appraisal Tendency Framework (Lerner and Tiedens, 2006) predicts that the emotion of fear is associated with greater risk aversion, while anger and happiness are correlated with greater risk taking. These propositions are supported by experimental studies (Lerner and Keltner, 2001; Kugler et al., 2012), in which emotions are induced prior to a risky choice task. Recent work by Nguyen and Noussair (2014), in which emotions are observed and tracked rather than induced, reports that fear, happiness, and anger all correlate positively with risk aversion, while emotional valence correlates negatively with risk aversion (negative emotions are associated with risk aversion).

Theoretical work, shows that those who are imprudent save less when their background risk increases (Kimball, 1990), behavior which may be financially hazardous for them as well as socially undesirable. Moreover, previous work has shown that imprudence correlates with poor decision-making (Noussair et al., 2014). In short, imprudent people get into financial trouble. It is, therefore, interesting and valuable to know what correlates with imprudent decision making. One factor that might get in the way of making good decisions are strong emotions. In this study, we consider which emotional states correlate with imprudent financial decisions. While research on the connection between emotions and risk aversion has established clear and important relationships, nothing is known about the correlation between emotional state and higher order risk attitudes. In this paper, we consider the relationship between prudent decision making and emotional state. Our design is guided by the theoretical work of Eeckhoudt and Schlesinger (2006) and the experimental implementation of Deck and Schlesinger (2010, 2014). Eeckhoudt and Schlesinger (2006) show how prudent and imprudent decisions can be distinguished using risk apportionment tasks that are simple to understand and straightforward to implement in the laboratory. Just as the willingness to accept a zero-mean risk can distinguish a risk averse from a risk seeking individual, a preference for accepting an unavoidable zero-mean risk in a relatively high, rather than a low, income state can reveal prudence. Even though this behavioral definition of prudence is model-free (just like the definition of risk aversion as a preference for the expected value of a lottery over the lottery itself is), a preference for assigning unavoidable risk to relatively high income states implies convex marginal utility or u”'(x) > 0, if one assumes that the DM maximizes expected utility (Eeckhoudt and Schlesinger, 2006).

We design and report an experiment that consists of two phases. In the first phase, participants are presented with a series of ten lotteries, in which two different payoff levels are equally likely. Each lottery is resolved after it is displayed. In the second phase of a session, subjects make choices between lotteries. The decisions have the feature that they offer a choice between two lotteries that are equivalent in terms of mean and variance, but that differ in skewness by varying whether they apportion risk to a high or low income state. We consider whether the emotional response to the presentation of the lotteries in the initial phase correlates with subsequent decisions. Additionally, we investigate correlations between some characteristics of individuals and their level of prudence. We measure our participants' cognitive ability using Raven's test of progressive matrices (Bors and Stokes, 1998) and personality traits as captured by the Big Five inventory (Gosling et al., 2003), and relate these to the decisions they make.

Our experiment shows that decisions depend on emotional state. The emotional state of participants in phase 1 of the experiment correlates with the level of prudence in their phase 2 decisions. More positive valence correlates with less prudent choices. Changes in arousal during the display of the prospects in the first phase of the experiment does correlate with decisions, with greater increases in arousal associated with more prudent choices. Our results as a whole indicate that stronger emotions tend to be associated with greater prudence, though all else equal, more positive emotional state correlates with less prudence. This pattern of results is similar to those observed by Nguyen and Noussair (2014) for risk aversion. They found that stronger emotions were correlated with more risk averse choices, and positive valence with less risk averse choices. We also observe that greater cognitive ability, as measured by the Raven's test score, is associated with greater prudence. This last result is in line with those reported by Noussair et al. (2014), using a different measure of cognitive ability, the Cognitive Reflection Test (Frederick, 2005). We also observe that conscientiousness correlates negatively with prudence.

## Materials and methods

### The participants and the setting

Eighty-three students from Tilburg University in the Netherlands participated in this computerized experiment, which was conducted at the CentER laboratory at Tilburg University in 2016^1^. There were six experimental sessions, each involving between 7 and 19 subjects. The majority of subjects studied economics. The average age was 22.5 years and 50.6% of the subjects were female.

The subjects were recruited among a pool of volunteers and were told that the experiment would last for up to 1 h. The experiment was programmed in Ztree (Fischbacher, 2007). The experiment consisted of four phases. At the start of each phase 1 to 3, separate instructions were read aloud. Instructions can be found online in the Data Sheet 1. During the experiment, facial expressions were recorded continuously by using video cameras. After completing the experiment, subjects were paid in private.

### Procedures and data gathered

In the first phase of the experiment, subjects were presented with 10 risky lotteries, displayed sequentially. Each lottery involved a 50/50 chance of receiving either a low or a high outcome with outcomes ranging from €1 to €13, and expected values ranging from €3.5 to €8.5. The lotteries displayed in phase 1 were unrelated to the lotteries that were presented later in the experiment.

After being presented on the screen, the lottery was resolved for each individual and the outcome of the lottery was then displayed on the screen for 10 s.^2^ Then, the next lottery appeared on the screen. The purpose of the first phase was to observe the emotional reaction caused by merely being exposed to risk and the emotional reaction caused by experiencing the outcome of the risky option. We register the emotion data at the time of presentation of the lottery itself, which we refer to as the *exposure* emotions. We also measure emotional state at the time each lottery is resolved and we refer to these as *feedback* emotions. In addition, we also retain for analysis the emotional state before the beginning of the experiment, and designate these as *initial* emotions.

The emotions are measured in the following manner. We videotape participants for the entire session with their consent. The videotapes are then analyzed with Noldus FaceReader™ software, which tracks facial expressions and analyzes the emotions they display. FaceReader has been employed in a number of experimental economics studies focusing on emotions (e.g., Breaban and Noussair, 2014; Nguyen and Noussair, 2014; Van Leeuwen et al., 2014; Habetinova and Noussair, 2015), but has also been used in marketing (Teixeira et al., 2012; Lewinski et al., 2014), and in psychological (Chentsova-Dutton and Tsai, 2010), research.

The FaceReader software tracks facial movements using the Facial Action Coding System, which associates specific muscle movements to the six basic universal emotions cataloged by Paul Ekman and his colleagues (e.g., Ekman et al., 1987; Ekman and Friesen, 2003). The emotions are happiness, fear, anger, disgust, surprise, and sadness. Facereader also measures how closely a facial expression conforms to a neutral state and generates an overall measure of emotional valence, as well as of arousal. The valence measure is calculated as Happiness—max{Anger, Fear, Sadness, Disgust}, that is, the value of the only positive emotion, happiness, minus the strongest of the four negative emotions. Arousal is a measure of emotional activation that varies from 0 to 1 and it is calculated as the average of the current highest five activation indicators corrected by a continuous average of activation during the last 60 s. The specific emotions are computed on a scale from 0 to 1, with one indicating complete conformity of facial movements to those associated with an emotion. It registers emotional state 30 times per second.

To compute the initial value of an emotion, we average the registered value of the emotion over the 60 s before phase 1 of the experiment began. During this period, subjects had no task to perform, and were passively waiting for the experiment to start. Exposure emotions represent the average over the 10 s during which a lottery is presented, and feedback emotions are computed as the average over the 10 s immediately following the resolution of the lottery.

**Figure 1:**
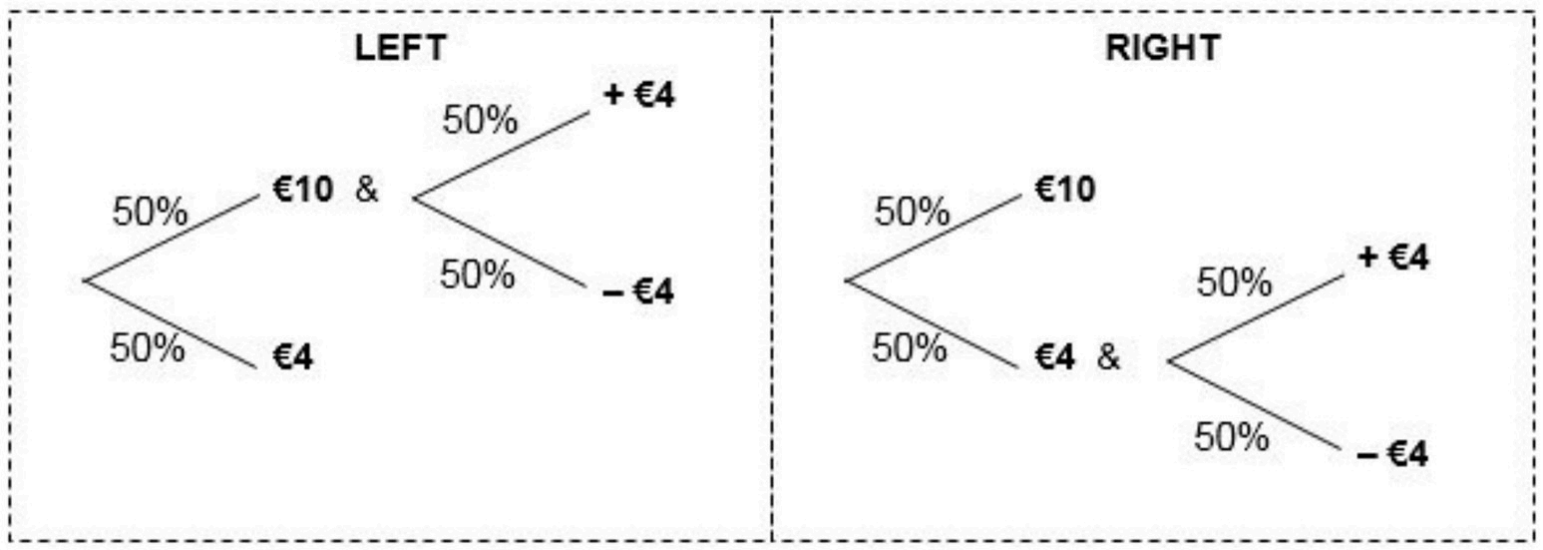
**Example of a choice from phase 2 of the experiment**.

The second phase of the experiment involves 10 direct pairwise choices. Each consists of a choice between one lottery that would be preferred by a prudent individual and an alternative that would be preferred by a decision maker who is imprudent. An example of a choice as presented to participants can be can be found in Figure 1. In both phases, all subjects were presented with all lotteries in the same order.

In the example of a choice shown in the figure, with 50% probability Left yields €10 and an additional 50/50 lottery yielding either a further gain or loss of €4. Otherwise, Left yields €4. Similarly, Right yields either €10 or €4 and an additional 50/50 lottery yielding either a gain of €4 or a loss of €4, both with 50% probability. Thus, the choice between left and right amounts to whether the subject prefers to apportion a zero-mean €4 risk to a state with relatively high wealth (left), or to a state with relatively low wealth (right). A choice for left (right) indicates that the decision maker can better cope with the zero-mean €4 risk when she has relatively more (less) wealth, implying that she is prudent (imprudent). The precise lotteries that were used are given in Table 1. In line with the existing literature (Deck and Schlesinger, 2010, 2014; Noussair et al., 2014), we use the number of prudent choices that a subject makes as a measure of the individual strength of prudence. If an individual chooses the prudent option in 6 or more of the 10 decisions she takes, we classify the individual as prudent. Analogously, if she chooses the prudent option in 5 or fewer instances, the individual is said to be imprudent.

**Table 1:** **Prudent lotteries used and choice proportions**.

**Choice #**	**Lottery displayed on left**	**Lottery displayed on right**	**% of instances in which prudent choice was made**
1	(10+(4_−4)_4)	(10_4+(4_−4))	88.0^***^
2	(6+(1_−1)_1)	(6_1+(1_−1))	79.5^***^
3	(12+(2_−2)_3)	(12_3+(2_−2))	79.5^***^
4	(9+(2_−2)_3)	(9_3+(2_−2))	74.7^***^
5	(8+(4_−4)_4)	(8_4+(4_−4))	83.1^***^
6	(6+(1_−1)_3)	(6_3+(1_−1))	73.5^***^
7	(7+(2_−2)_2)	(7_2+(2_−2))	85.5^***^
8	(11+(3_−3)_3)	(11_3+(3_−3))	88.0^***^
9	(13+(4_−4)_4)	(13_4+(4_−4))	85.5^***^
10	(12+(2_−2)_2)	(12_2+(2_−2))	86.7^***^

(x_y) indicates a lottery with an equal probability of receiving either x or y; outcomes in euros;

*** indicates significant difference at 1% level from random choice between left and right option, binomial test, two-sided.

In the third phase of the experiment, cognitive ability is measured using Raven's advanced progressive matrices test (Raven et al., 1998), a protocol commonly used to measure fluid intelligence. The task involves choosing the correct one out of eight possible alternatives to complete a 3-by-3 matrix of abstract symbols in a consistent pattern. Due to the limited amount of time available in our sessions, we used the short form of the test proposed by Bors and Stokes (1998) that consists of 12 tasks. Subjects were given a total of 10 min to complete the 12 tasks, and were allowed to revise previous answers if time allowed.

The final phase of the experiment consists of a questionnaire designed to obtain a classification of personality. More specifically, we administer the 10-item Big Five personality measure developed by Gosling et al. (2003). This measure allows one to classify individual differences in personality into five broad dimensions: extraversion, agreeableness, conscientiousness, neuroticism, and openness to new experiences, by registering applicability of 10 items regarding subject's personality on a scale from 1 (disagree strongly) to 7 (agree strongly). In addition, background information of subjects regarding age, gender, study, year of study was gathered. There is some previous evidence that the dimensions of openness and extraversion correlate negatively with risk aversion, and neuroticism, agreeableness and conscientiousness correlating positively (Nicholson et al., 2005; Becker et al., 2012). We are unaware of any prior work correlating personality characteristics and prudence.

**Figure 2:**
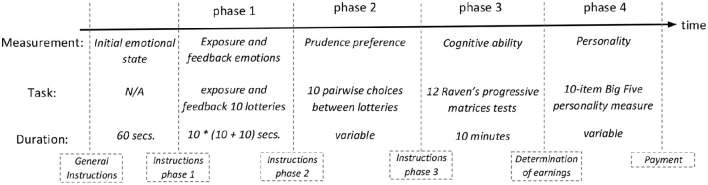
**Timing of Experiment**.

Thus, for each participant, we observe the emotional reaction caused by being exposed to risk and the emotional reaction caused by experiencing the outcome of a risky lottery (phase 1), as well as a measure of the degree of prudence (phase 2), of cognitive ability (phase 3), and of personality dimensions (phase 4). Figure 2 below shows a timeline of the experiment.

To avoid potential income effects on the measure of prudence [such as Thaler and Johnson's (1990) house money effect] and to provide incentives for truthfully reporting preferences, the random incentive mechanism was used. That is, subjects were informed from the outset that at the end of the experiment, phase 1 or phase 2 would be randomly selected with equal probability. If the first phase is selected, the observed outcome of one of the ten of the lotteries (randomly selected) count toward the participant's earnings. If the second phase is selected, the computer randomly selects one of the ten pairs of lotteries. The outcome of the chosen lottery in that pair would then count toward earnings. On top of these earnings, subjects received €0.50 for each of the correct answers to the Raven test in phase 3 as well as a fixed participation fee of €2. On average, subjects earned €12.18 during the experiment.

One of our design choices merits some further comment. We have chosen to track, without attempting to influence, the emotions and arousal level that our participants exhibit during our task. An alternative would be to induce different emotional or arousal states and compare the resulting decisions, as many other authors have done. The induction of emotions is well suited to addressing questions regarding the causal effects of emotional variables on decision making, and is a powerful tool for addressing many if not most important questions in emotion research. The design we have chosen is meant to document correlates of prudent decision making, rather than causal relationships. We consider whether those who tend to exhibit particular emotions, greater or less arousal, and positive or negative emotional state after exposure to and experience with lotteries, exhibit more or less prudence in subsequent decisions. Identifying such correlates of prudence in decision making is the purpose of this research.

## Results

A clear majority of individuals in the study were prudent. 42.17% (35 of 83) of participants made a prudent decision at every opportunity. Another 46.99% (39 of 83) made a prudent choice between 6 and 9 times, indicating that they chose prudently in a majority of instances in which they had an opportunity to do so. Thus, 89.16% of individuals are classified as prudent. 10.84% (9 of 83) of participants made fewer than 6 prudent choices are thus classified as imprudent. The fact that a majority of participants is prudent is consistent with the previous literature (Deck and Schlesinger, 2010, 2014; Ebert and Wiesen, 2011, 2014; Noussair et al., 2014).

**Figure 3:**
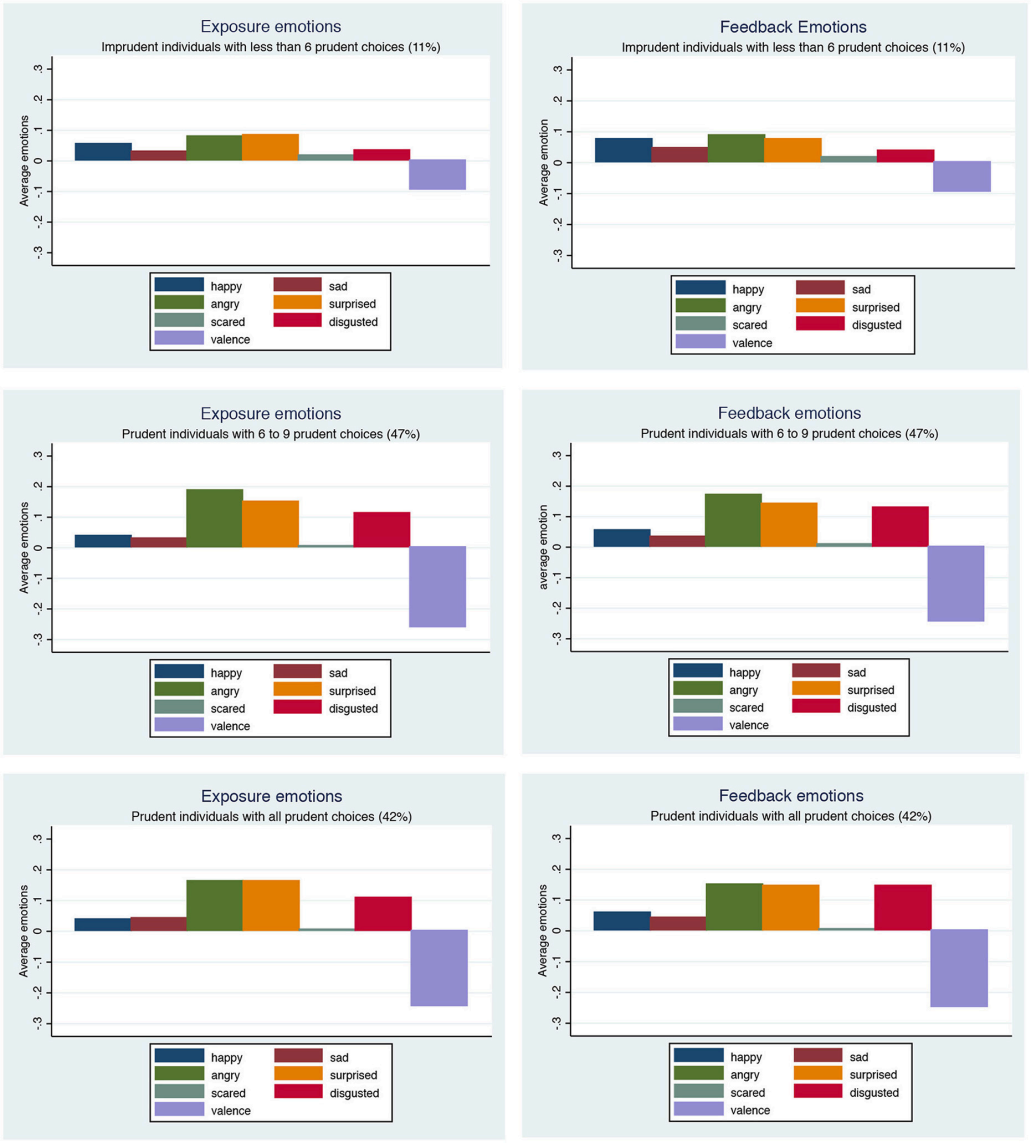
**Emotional profiles and the number of prudent decisions**.

Figure 3 illustrates the average emotional state in phase 1 of the experiment for those who made 0–5, between 6 and 9, and who made 10 prudent decisions in phase 2. The panels on the left indicate the average value of the exposure emotions, measured at the time that the lotteries are displayed in phase 1. Those on the right are the feedback emotions, those registered at the time that each of the phase 1 lotteries is resolved. The strength of the various emotions is typically similar at the exposure as at the feedback point. The figure shows that those who exhibit more negative valence, as well as stronger anger, surprise and disgust, and lower happiness, when viewing the lotteries, make more prudent decisions. The results are similar whether exposure or feedback emotions are considered.

To make these impressions more precise and to control for other potential influences on prudence, we conduct Poisson count regressions in which the number of prudent choices is the dependent variable. The estimates for feedback emotions are reported in Table 2, and those for exposure emotions are in Table 3.^3^

**Table 2:** **Number of prudent choices as a function of emotional, ability, and personality measures; feedback emotions**.

	**(1)**	**(2)**	**(3)**	**(4)**	**(5)**	**(6)**	**(7)**
Gender	0.024	0.014	0.003	0.021	0.031	0.017	0.029
Arousal		−0.258	0.283^*^	−0.276	−0.252		
Valence	−0.086^**^	−0.102^**^	0.039	−0.090^**^	−0.108^***^		
Raven score		0.026^***^	0.026^***^		0.026^***^		0.027^***^
Extraverted					0.012		0.019^*^
Agreeableness					−0.005		−0.009
Neuroticism					0.009		0.006
Conscientiousness					−0.027^***^		−0.028^***^
Openness to experiences					0.026^**^		0.018
Happy						0.066	−0.010
Sad						0.086	0.212
Scared						−0.319	−0.609
Angry						0.121^**^	0.082
Disgusted						0.327^***^	0.360^***^
Surprised						0.191^***^	0.153^***^
	Obs 770	Obs 770	Obs 761	Obs 770	Obs 770	Obs 770	Obs 770
	Groups 10	Groups 10	Groups 10	Groups 10	Groups 10	Groups 10	Groups 10

Dependent variable is the number of prudent decisions [0, 10] made by an individual in phase 2 of the experiment. In all equations other than (3), the emotion and arousal variables are those averaged over the 10 s after the resolution of the 10 lotteries in phase 1. In Equation (3), the emotion and arousal variables are the difference between those in the 60 s before the start of phase 1 and those at the time of the resolution of the lotteries. Regressions use panel data format that adjusts the standard errors for repeated measures.

^*^, ^**^, ^***^ denotes significance at the 10%, 5%, 1% level.

**Table 3:** **Number of prudent choices as a function of emotional, ability, and personality measures; exposure emotions**.

	**(1)**	**(2)**	**(3)**	**(4)**	**(5)**	**(6)**	**(7)**
Gender	−0.0004	−0.008	−0.015	−0.0004	0.006	−0.012	−0.003
Arousal		−0.016	0.279^**^	−0.006	−0.071		
Valence	−0.055	−0.054	0.026	−0.055	−0.063^*^		
Raven score		0.025	0.026^***^		0.026^***^		0.027^***^
Extraverted					0.020^*^		0.023^**^
Agreeableness					−0.005		−0.006
Neuroticism					0.006		0.004
Conscientiousness					−0.025^**^		−0.027^***^
Openness to experiences					0.019		0.015
Happy						−0.085	−0.114
Sad						0.167	0.242^*^
Scared						−0.150	−0.341
Angry						0.066	0.047
Disgusted						0.217^***^	0.238^***^
Surprised						0.160^***^	0.131^***^
	Obs 720	Obs 720	Obs 714	Obs 720	Obs 720	Obs 720	Obs 720
	Groups 10	Groups 10	Groups 10	Groups 10	Groups 10	Groups 10	Groups 10

Dependent variable is the number of prudent decisions [0, 10] made by an individual in phase 2 of the experiment. In all equations other than (3), the emotion and arousal variables are those averaged over the first 10 s that the 10 lotteries in phase 1 are displayed. In equation (3), the emotion and arousal variables are the difference between those in the 60 s before the start of phase 1 and those at the time of the display of the lotteries. Regressions use panel data format that adjusts the standard errors for repeated measures.

^*^, ^**^, ^***^ denotes significance at the 10%, 5%, 1% level.

In results 1–4, we report our results concerning the correlates of prudence. The first result below indicates that there is a negative correlation between the overall valence of emotional state and prudence. Those in a more positive emotional state are less prudent.

### Result 1: positivity of emotional state, when facing risky lotteries, correlates with imprudence

#### Support for result 1

Table 2 contains estimates of Poisson count regressions in which the number of prudent choices is the dependent variable. The valence variable is evaluated at the feedback stage. The coefficients of valence in specifications (1), (2), (4), and (5) indicate that valence is a significant predictor of decisions. In all four regressions, the coefficient of valence is negative and significant at the *p* < 0.05 level in three specifications and *p* < 0.01 level in one specification. Those in a more positive state are more imprudent, while more negative states are associated with prudence. In Table 3, we report the results from similar regressions with valence measured at the exposure stage. In all four specifications in which it appears, the variable Valence is negative in sign, though it is marginally significant only in specification (5). Overall, in our view, the balance of the evidence indicates a negative relationship between positivity of emotional state and prudence.^4^

The second dimension of emotional state that we consider is arousal. While positive emotional state is associated with less prudence, we find that stronger arousal is associated with greater prudence. However, as we describe in the supporting argument for result 2, it is the change in arousal from the initial level that is correlated with subsequent decisions. The level of arousal at the time of exposure to or feedback from the lotteries in phase 1 is uncorrelated with the number of prudent choices in phase 2.

### Result 2: increases in arousal when facing risky lotteries correlates with prudent decision making

#### Support for result 2

Specifications (2), (4), and (5) in Tables 2, 3 reveal that the absolute amount of arousal in phase 1 is not correlated with prudence in decision making. However, as specification (3) shows, the results are different if changes in arousal from the beginning of the session to the moment of measurement are considered. In equation (3), the emotional variables are the actual value of the emotion at the moment of feedback or exposure in phase 1, minus the initial level at the beginning of the session prior to the start of phase 1. In both tables, the results show that overall arousal level does not presage more prudent decision making, but an increase in arousal when confronted with risky lotteries does correlate with a greater number of prudent choices.

We now turn to the individual emotions as correlates of decisions. The principal pattern in the data is that more intense emotions, in particular surprise and disgust, correlate with greater prudence. There is some evidence that greater anger and sadness also are associated with more prudence. Fear and happiness do not exhibit a significant relation with prudent decision making. Our findings are reported as result 3.

### Result 3: stronger emotions are correlated with greater prudence

#### Support for result 3

The results are shown in specifications (6) and (7) in Table 2 for emotions in the feedback stage and in Table 3 for the exposure stage. The tables reveal a significantly positive relationship between disgust and surprise with the number of prudent decisions made in all relevant equations. Sadness and anger are each significant in one of the four specifications in which they appear. In all cases, a greater value of the emotion correlates with greater prudence.

The last result considers the other correlates of prudence that our design permits us to evaluate.

### Result 4: there are no gender differences in the average level of prudence. prudence is positively correlated with cognitive ability. prudence is negatively correlated with conscientiousness

#### Support for result 4

In all of the specifications reported in Tables 2, 3, the variable Gender is insignificant. The variable Raven, the score of an individual on the Raven's test, is significant at the 1% level in all estimated equations in which it appears. Furthermore, none of the big 5 personality traits is significant other than conscientiousness.

## Discussion

We observe that those who experience more positive valence at the time of the resolution of risky lotteries tend to make less prudent subsequent decisions. The same correlation obtains if valence at the time of presentation of the lotteries is considered, although this effect is only marginally significant. This result is similar in spirit to those obtained for risk aversion by a number of authors, who find that negative emotional state is associated with greater risk aversion. There are a number of possible explanations for this correlation. If a negative emotional state prompts more pessimistic beliefs, as under the Affective Generalization Hypothesis, an individual with negative valence might believe that the bad state is more likely to occur than the good state. If this is the case, and the agent is risk averse, she will apportion an unavoidable zero-mean risk to what she believes is the less likely state, i.e., the one yielding the relatively high outcome. Alternatively, it may be the case that a negative emotional state prompts individuals to behave defensively by maximizing their minimum payoff. This pattern would translate into declining to accept zero-mean risks when given an opportunity to do so (risk aversion), and apportioning unavoidable risks into relatively high income states when possible (prudence). Future research would be needed to distinguish between the hypotheses that a negative emotional state leads individuals to apply a heuristic in which they maximize their minimum payoff and the alternative that negative emotions prompt more risk averse as well as more prudent decisions.

We also observe that increases in arousal during the phase 1 task, which can be interpreted as integral arousal, is positively correlated with prudence in subsequent decisions. It may be the case that greater arousal, like more negative valence, leads to more pessimistic beliefs. The consequence would be that the high income state is viewed as less likely, and that a risk averse individual would allocate the risk to what she believes is the less likely state, and generate behavior consistent with prudence. Alternatively, arousal may lead to a focus on relatively unfavorable outcomes and choices that maximize payoff under the worst possible outcome. While some prior research associates greater arousal with risk taking (Haim, 1994), other work argues that underarousal increases risk taking as individuals seek arousing stimuli (Schmidt et al., 2013). Here, it may be the case that underaroused individuals place the risk in the low income state as stimulation to increase their level of emotional arousal.

An overall pattern emerges with respect to the relationship between individual emotions and prudence in decision making. This is that stronger emotions are associated with more prudent decision making. The result is also similar to, and might be viewed as somewhat of an extension of, those reported by Nguyen and Noussair (2014), who also find that stronger emotions correlate with risk aversion, though they observe their relationship for a different set of emotions. Explaining why there is a relationship between more intense emotions and prudence is beyond the scope of what this experiment can test, but the explanations may be similar to those proposed for the correlation between prudence and valence or arousal described above. Strong emotions might influence beliefs about the likelihood of each state or encourage the use of heuristics such as the maximization of minimum payoff.

The absence of a gender effect and the strong link between prudence and cognitive ability echoes the results of Noussair et al. (2014), who observed the same patterns in a large demographically representative sample of the Dutch population. The emerging pattern with regard to gender differences in prudence contrasts with that for risk aversion, in which gender differences are widely observed (see e.g., Eckel and Grossman, 2008). The particular relationship we observe between personality and prudence is surprising for a couple of reasons. The first reason is that the Big Five personality characteristics and risk aversion exhibit a pattern of correlation that is both strong and intuitive to interpret. Here, the relationship is relatively weak with only conscientiousness exhibiting a robust relationship. The second reason is that because prudence is associated with high cognitive ability and precautionary savings, one might think that it would also be correlated with greater conscientiousness, rather than less, as we observe here. However, the effect of conscientiousness remains in regressions (not reported here but available from the authors), in which Raven's score is left out of the specification. The effect of conscientiousness becomes insignificant when the emotional state variables of valence, arousal, and specific emotions are not included in the specification, suggesting that emotional states may affect individuals' decisions differently, depending on their personality profile. Conducting an analysis of the mediating and moderating relationships of such a large number of personality characteristics and emotional variables on prudence would require a much larger data set than we gathered for this study, but we believe it would be worthwhile to pursue such an analysis in future work.

## Author contributions

All authors listed, have made substantial, direct and intellectual contribution to the work, and approved it for publication. All authors contributed equally; authors names appear in alphabetical order.

## Funding

We thank the VIDI program of NWO for funding to support this experiment.

### Conflict of interest statement

The authors declare that the research was conducted in the absence of any commercial or financial relationships that could be construed as a potential conflict of interest.

## Footnotes

^1^Tilburg University, where the experiment was conducted, does not have an Institutional Review Board. This is fully in line with Dutch law, which does not require IRB review for social science research. Subjects gave verbal consent to be videotaped. However, they were unaware that their facial expressions would be analyzed.

^2^When single emotions occur and there is no reason for them to be modified or concealed, expressions typically last between 0.5 to 4 seconds and involve the entire face (Ekman, 2003). The onset and offset of a sincere emotional response in reaction to a stimulus is generally between 2/3 of a second and 4 seconds (Hager and Ekman, 1985; Hess and Kleck, 1990). Thus, the 10 second window that we study should capture the full reaction to exposure to the lottery or to feedback from the lottery outcome. The relatively long time horizon in which we measure emotional state at the beginning of the experiment, allows us a relatively large amount of data on subjects' initial mood at the outset of the session.

^3^Subjects were told to pay attention to their screen and were asked not to touch their face during the experiment. This ensured that we were able to gather facial expression data for the vast majority of decisions. There are 60 to 69 for missing observations for the results in Table 2 and 110 to 116 missing observations for the results reported in Table 3. These missing observations are instances when subjects looked away from their computer screens or covered part of their faces with their hands.

^4^We also considered whether the difference in valence at the time of feedback, between instances of positive and negative outcomes of the lottery, predict prudence in decision making in phase 2. It is presumed that individuals will tend to have more positive valence after a favorable than an unfavorable outcome. However, for those who have a relatively high value of the difference, Valdiff = Valence(Favorable outcome) − Valence(Unfavorable outcome), might be more prudent. This is because, if a positive emotional state leads to more risk taking, and a negative emotional state leads to lower risk taking, individuals with a relatively high value of Valdiff might be more willing to apportion the unavoidable risk to the high income state. This would lead to a positive correlation between Valdiff and prudent decision making. However, no such correlation appears in the data.
