# Supplementary material for: Prudence, Emotional State, Personality, and Cognitive Ability
Source: Front Psychol. 2016 Oct 28;7:1688. doi: 10.3389/fpsyg.2016.01688 (PMC5083905; doi:10.3389/fpsyg.2016.01688)
Supplement: Supplementary file 1 [file DataSheet1.docx]

**Appendix A: Experimental Instructions**

<General Instructions >

Welcome to this experiment on decision making. Once the experiment has started, you are not allowed to talk to the other participants. You are requested to turn off you mobile phone and raise your hand if you have any questions about the experiment. Your question will then be answered in private by the experimentalist. During the experiment you will be videotaped with the webcam located on top of your screen. After reading the general instructions, the experimentalist will turn on the webcam and a video will be recorded throughout the entire session. The only purpose of the videotape is research and it will not be viewed by anyone else than the experimentalist. Your personal information and video will be confidential.

This experiment involves three parts and a short questionnaire. At the beginning of each part, the experimenter will hand out part-specific instructions and read these instructions out aloud. For now, it is important to know that at the end of the experiment, i.e., once everybody has completed all parts and has filled in the questionnaire, the computer will select 1 of the first 2 parts at random (i.e., both parts have an equal chance to be selected by the computer). From this part, the outcome of 1 task will randomly be selected to count towards your earnings. In addition, you will receive the outcome of part 3 and a show-up fee of €2. Thus, your total earnings in this experiment are determined as follows:

Total Earnings = outcome of a randomly selected task from part 1 or part 2 + outcome of part 3 + €2

If you have any question at this point, please raise your hand to be helped in private. If there are no questions, the experimenter will hand out the instructions of the first part of the experiment.

Good luck!

<Instructions Part 1>

Part 1 consists of 10 tasks. Each task concerns an option that yields a monetary outcome depending on chance. An example option is given below. This example is not an option that you will actually encounter during the experiment, but merely illustrates the format.


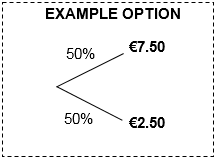


As you can see, the example option yields either €7.50 or €2.50, both with 50% probability. In the experiment, the outcome of each option will directly be determined by the computer. For that purpose, you are asked to press the “proceed” button depicted below each option. Then, the outcome of the option (in the example €7.50 or €2.50) will be displayed and a next task will start. In fact, since we will wait for everybody to complete each task, you might have to wait a while before you proceed to the next task. As explained, if this part is selected by the computer at the end of the experiment, 1 of the 10 tasks (randomly chosen) will count towards your earnings. Please raise your hand if you need further explanation from the experimenter. If there are no questions, the experimenter will soon start the program.

<Instructions Part 2>

Part 2 consists of 10 tasks. Each task concerns a choice between two options labelled Left and Right. Both options yield an amount of euros depending on chance. An example choice is given below. This example is not a choice that you will actually encounter during the experiment, but merely illustrates the format.

**Example Choice**Please make a choice between Left and Right:

**LEFT**

**– €2.50**

**+ €2.50**

50%

50%

**€2.50**

**€4.50** &

50%

50%

**RIGHT**

**– €2.50**

**+ €2.50**

50%

50%

**€2.50** &

**€4.50**

50%

50%


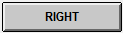


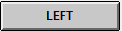


As you can see, in the example choice, option Left yields €2.50 with 50% probability or - also with 50% probability - option Left yields €4.50 *and* another option yielding either an additional gain of €2.50 or additional loss of €2.50, both with 50% probability. Thus, option Left yields €2.50 with 50% probability, €7.00 (€4.50 + €2.50) with probability 25% (50%*50%) or it yields €2 (€4.50 - €2.50) with probability 25% (50%*50%). Similarly, option Right yields €4.50 with 50%, €5.00 (€2.50 + €2.50) with 25% (50%*50%) or it yields nothing (€2.50 - €2.50) with 25% (50%*50%) probability. Note that the expected outcome of both options in the example is the same. However, the potential outcomes, and the chances to get these outcomes, differ between the options. This will be the case for all tasks that you will encounter in part 2. The way you complete each task is by clicking the button corresponding to the option that you prefer.

As explained, if this part is selected by the computer at the end of the experiment, 1 of the 10 tasks (randomly chosen) will count towards your earnings. That is, the outcome of the option that you have chosen will be determined by the computer and that outcome will count towards your earnings. Thus, it is important that you choose carefully between the options, since there is no possibility to revise your decisions. Please raise your hand if you need further explanation from the experimenter. If there are no questions, the experimenter will soon start the program.

<Instructions Part 3>

Part 3 concerns a test of observation and clear thinking that consists of 12 tasks. The result of the test is strictly confidential and anonymous. Each correct answer will earn you €0.50. Firstly, we will consider an example task followed by explanations of the answers to it. This is intended to show you how the test works, or, if you have seen tests of this sort before, to remind you how they work.

**Example task**

**Please identify the element that completes the pattern below:**


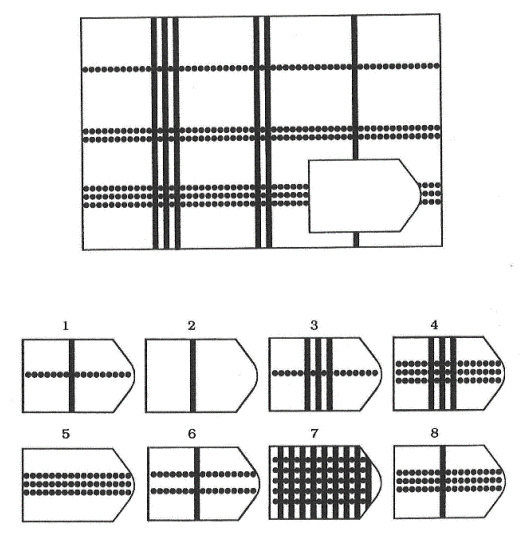


As you can see, in the example task, you are asked to complete the pattern by selecting 1 of the 8 elements depicted below the pattern. The correct answer in this case is element 8, since it is the only element that correctly completes the pattern going across the row and down the column. As you can see:

- Element 1, 2 and 6 complete the pattern of one solid line going down the column, but do not complete the pattern of three dotted lines going across the row.
- Element 4 and 5 correctly complete the pattern of three dotted lines going across the row, but do not complete the pattern of one solid line going down the column.
- Element 3 and 7 do not complete the pattern of three dotted lines going across the row, and do not complete the pattern of one solid line going down the column.

Each of the 12 tasks will be similar to the example task, except that they get progressively more difficult. You have 10 minutes to finish all 12 tasks. If you did not complete all tasks after 10 minutes, the unanswered ones will be taken as wrong answers by the computer.

The tasks will be presented on a screen as shown below:


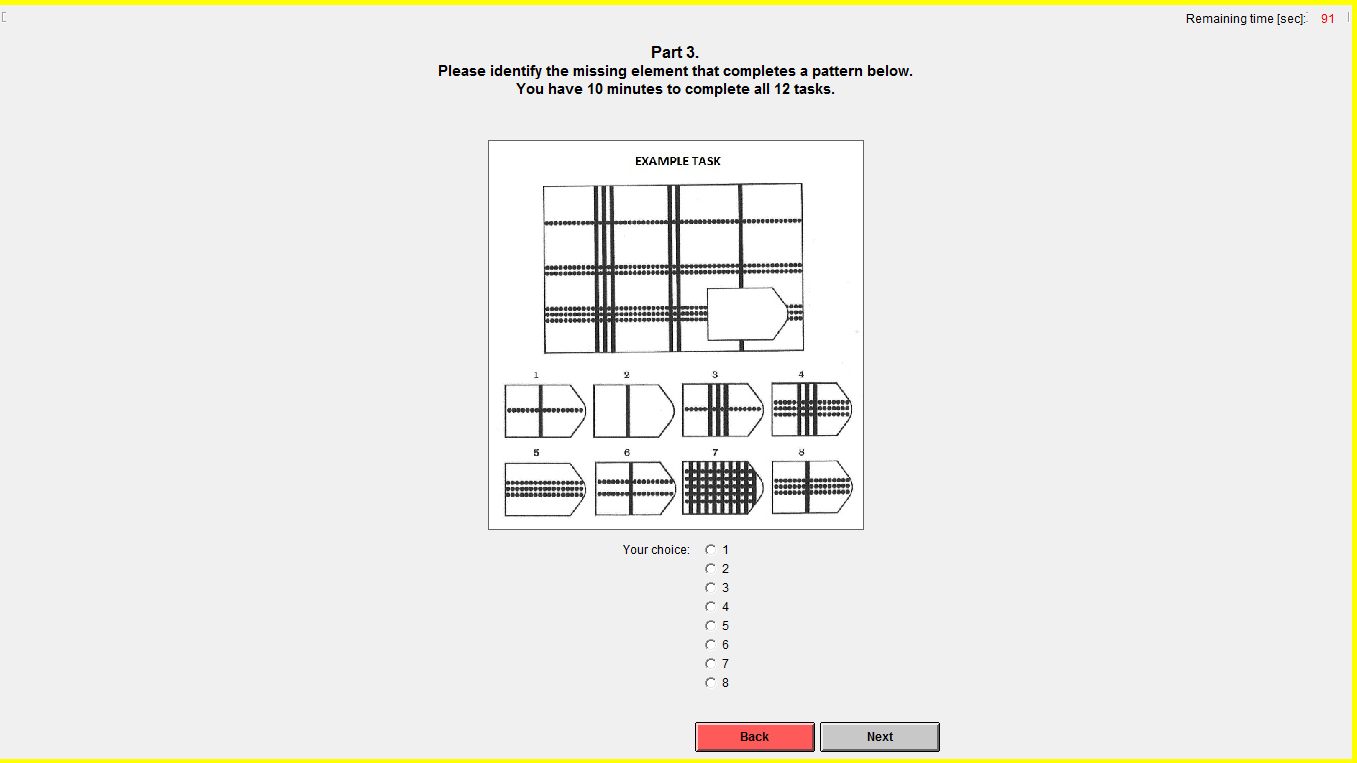


As you can see, the pattern and elements are presented and you are asked to select the element that completes the pattern at the bottom of the screen. To register your answer, you must click the radio button on the bottom of the screen and click the Next button. Note that if you do not make a choice, the computer will treat this question as unanswered and therefore take it as a wrong answer. Also note that in this part of the experiment, you are free to go back and forth with the Next and the Back button to review your answers if you still have time. The remaining time in seconds is always shown at the top of the screen.

As explained, each correct answer in this part will earn you €0.50 which will be added to your earnings from the previous parts. Please raise your hand if you need further explanation from the experimenter. If there are no questions, the experimenter will soon start the program.
